# *Ophiocordyceps zhenxingensis* sp. nov. and its microbiota during sexual and asexual stages in nature

**DOI:** 10.1128/spectrum.02159-24

**Published:** 2025-07-11

**Authors:** Huiling Tian, Gangxiang Fei, Jingqiang Guan, Zhongshun Xu, Jiaojiao Qu, Yongdong Dai, Xiao Zou

**Affiliations:** 1 Institute of Fungus Resources, College of Life Sciences, Guizhou University 71206, Guiyang, Guizhou, China; 2 College of Tea Sciences, Guizhou University 71206, Guiyang, Guizhou, China; 3 Fungus Medicine Research Center/School of Basic Medical Science, Guizhou University of Traditional Chinese Medicine 326770 , Guiyang, Guizhou, China; 4 Key Laboratory of Plant Resource Conservation and Germplasm Innovation in Mountainous Region, Guizhou University 71206, Guiyang, Guizhou, China; College of Life Sciences, Nanchang University, Nanchang, Jiangxi, China

**Keywords:** new species, *Ophiocordyceps zhenxingensis*, sexual and asexual stages, phylogenetic analyses, community composition, function predictions

## Abstract

*Ophiocordyceps* holds significant importance in forest arthropod population regulation, biological control of agricultural pests, and the development of bioactive substances. Investigating the microbial community composition of wild *Ophiocordyceps* is significant for understanding its individual development process, understory cultivation, and taxonomy. In this study, a novel insect pathogenic fungus, *Ophiocordyceps zhenxingensis*, parasitizing Hymenoptera larvae, originating from Liaoning Province, China, was delineated based on morphology and phylogenetics. The microbial communities in the sclerotium and host surface complex of its different reproductive stages (sexual and asexual) were investigated. *O. zhenxingensis* was detected within the *Hirsutella citriformis* subclade, being closely related to *Hirsutella gigantea* and *Ophiocordyceps elongata*. It was morphologically characterized by solitary or multiple stromata, semi-submerged perithecia, and viscous basidiospores. Proteobacteria, Actinobacteriota, and Bacteroidota were the major bacterial taxa, and Basidiomycota and Ascomycota were the major fungal taxa. *Tomentella*, *Sebacina*, and *Russula* were abundant in the host surface complex that belongs to mycorrhizal fungi. The microbial co-occurrence network suggested that microbial relationships were predominantly positive correlations, and positive correlations were more pronounced in the asexual stage than the sexual stage. The microbial co-occurrence network of *O. zhenxingensis* was more complex during the asexual stage; it exhibited greater metabolic activity during the asexual stage. These results have enhanced our understanding of the composition, diversity, and functions of the endophytic microbial community of *Ophiocordyceps*, furnishing additional evidence for classifying its sexual and asexual stages and establishing a theoretical basis for subsequent research on its individual development.

**IMPORTANCE:**

*Ophiocordyceps* exhibits two distinct life stages (asexual and sexual) in its natural environment. The symbiotic microorganisms associated with the fungus play a crucial role in its growth and development. We have identified a new species, *Ophiocordyceps zhenxingensis*, which belongs to the *Hirsutella citriformis* subclade. A large number of mycorrhizal fungi were found in the insect appendages of *O. zhenxingensis*, whereas the fungal community within the sclerotium was predominantly composed of Ascomycota. During the asexual stage, *O. zhenxingensis* exhibited greater microbial diversity and stronger positive correlations among microorganisms. Additionally, it possesses a richer array of metabolic pathways. These results have deepened our knowledge of the composition, diversity, and roles of the microbial community in *Ophiocordyceps*, providing further evidence for distinguishing its sexual and asexual stages and laying a theoretical foundation for future research on its developmental processes.

## INTRODUCTION

*Ophiocordyceps* (Hypocreales, Ophiocordycipitaceae), an entomopathogenic fungal genus, was established in 1931 based on the type species *Ophiocordyceps blattae* Petch (1). Kobayasi (2) and Mains (3) classified *Ophiocordyceps* as *Cordyceps sensu lato*, but it was subsequently reinstated as a separate genus based on morphological and multigene phylogenetic analyses (4). More than 330 species names have been accepted for the genus (https://www.indexfungorum.org/, 16 April 2024) (5, 6).

*Ophiocordyceps* spp. are of significant value in arthropod population control, maintenance of ecosystem balance, and the development of medicines and bioactive substances (7). *Hirsutella rhossiliensis* Minter and Brady (1980) and *Hirsutella minnesotensis* Chen, Liu, and Chen (2000) are highly invasive and lethal to plant-parasitic nematodes (8). *Hirsutella thompsonii* Fisher exerts control over the two-spotted leaf mite and was once sanctioned by the U.S. Government as a major acaricide (9). *Ophiocordyceps sinensis* (Berk.) Sung et al. is a traditional Chinese medicine with effects of toxifying the lungs and kidneys, staunching bleeding, resolving phlegm, and alleviating cough and asthma (10). Nevertheless, the excessive exploitation of *O. sinensis* has not only resulted in an increasing scarcity of its wild resources but also triggered concerns about its ecological environment. In 2018, *O. sinensis* was listed as a “vulnerable” species on China’s Red List of Biodiversity (11).

The wild *Ophiocordyceps* species complex is formed by various microorganisms in conjunction with a host insect (12–14). These microbiotas play a crucial role in nutrient cycling, infection of the insect host, and growth and maturation of sclerotia and stromata (15, 16). The fungal dynamics and diversity associated with *Ophiocordyceps* in the soil regulate the fungal-larval relationship and impact the growth and development of *Ophiocordyceps* (17, 18). Some species in the outer cortex of the host surface complex may facilitate fungal hyphal formation (19). Fungi reproduce by generating a network of hyphae and secreting enzymes that convert complex nutrients into monosaccharides for cell growth (20). Li et al. infested ghost moth larvae with multiple species from its intestinal fungal complex combined with *Hirsutella sinensis* conidia, resulting in increased infestation efficacy and shortened larval stiffening time (10). Zhang et al. demonstrated that *Trichoderma*, *Archaeorhizomyces*, *Sebacina*, entomopathogenic fungi, and some bacterial species play important roles in the ontogeny and maturation of *O. sinensis* (19).

*Ophiocordyceps* can exist in two entirely distinct lifestyles in its natural environment. Specifically, in its life history, there is an asexual reproduction stage characterized by asexual spore formation (conidia) or the absence of spore formation, and a sexual reproduction stage characterized by the formation of stromata (or its absence) and sexual spore production (ascospores). The former stage is called the anamorph, and the latter is called the sexual stage (teleomorph) (12). The asexual and sexual stages of *Ophiocordyceps* have always commanded considerable attention in taxonomy, and the results are mainly based on morphology and phylogeny based on DNA sequences (4, 21–24). Nevertheless, the companion microorganisms of *Ophiocordyceps* are equally indispensable for its growth and development. The difficulty in obtaining large amounts of *Ophiocordyceps* sexual stages in nature resulted in the scarcity of research on the microbial community composition of its two distinct reproductive stages (asexual and sexual).

The sexual and asexual stages are wholly distinct reproductive stages. Furthermore, the sclerotium and host surface complex are two disparate parts, with the former being the host tissues infected by the fungus and the latter connecting the host with the soil, which contains the insect cocoon, mycelia, and a few soil components in a complex mixture. Consequently, it was hypothesized that the sexual and asexual stages and the sclerotium and host surface complex possess unique microenvironments and have different microbial compositions and functions. Symbiotic microorganisms exert a crucial role in the growth and maturation of *Ophiocordyceps* fruiting bodies (15, 19). Certain microorganisms combine with insect pathogenic fungi to enhance host infection, thereby facilitating the formation of *Ophiocordyceps* individuals. The disparities between the sexual and asexual stages might also be associated with these symbiotic microorganisms. Based on the above research background, in this study, a new *Ophiocordyceps* species parasitizing (Hymenoptera) larvae, including sexual (50 plants; Fig. S1) and asexual (80 plants; Fig. S2) wild specimens, were collected from Liaoning Province and identified through multigene association and morphological studies. High-throughput sequencing was utilized to explore the microbial community composition and diversity of the sexual and asexual stages of sclerotium and host surface complex. A distinct comprehension of the composition or differences in their respective symbiotic microorganisms will facilitate the understanding of the formation mechanism of the sexual and asexual stages.

## RESULTS

### Phylogenetic analyses

**Fig 1:**
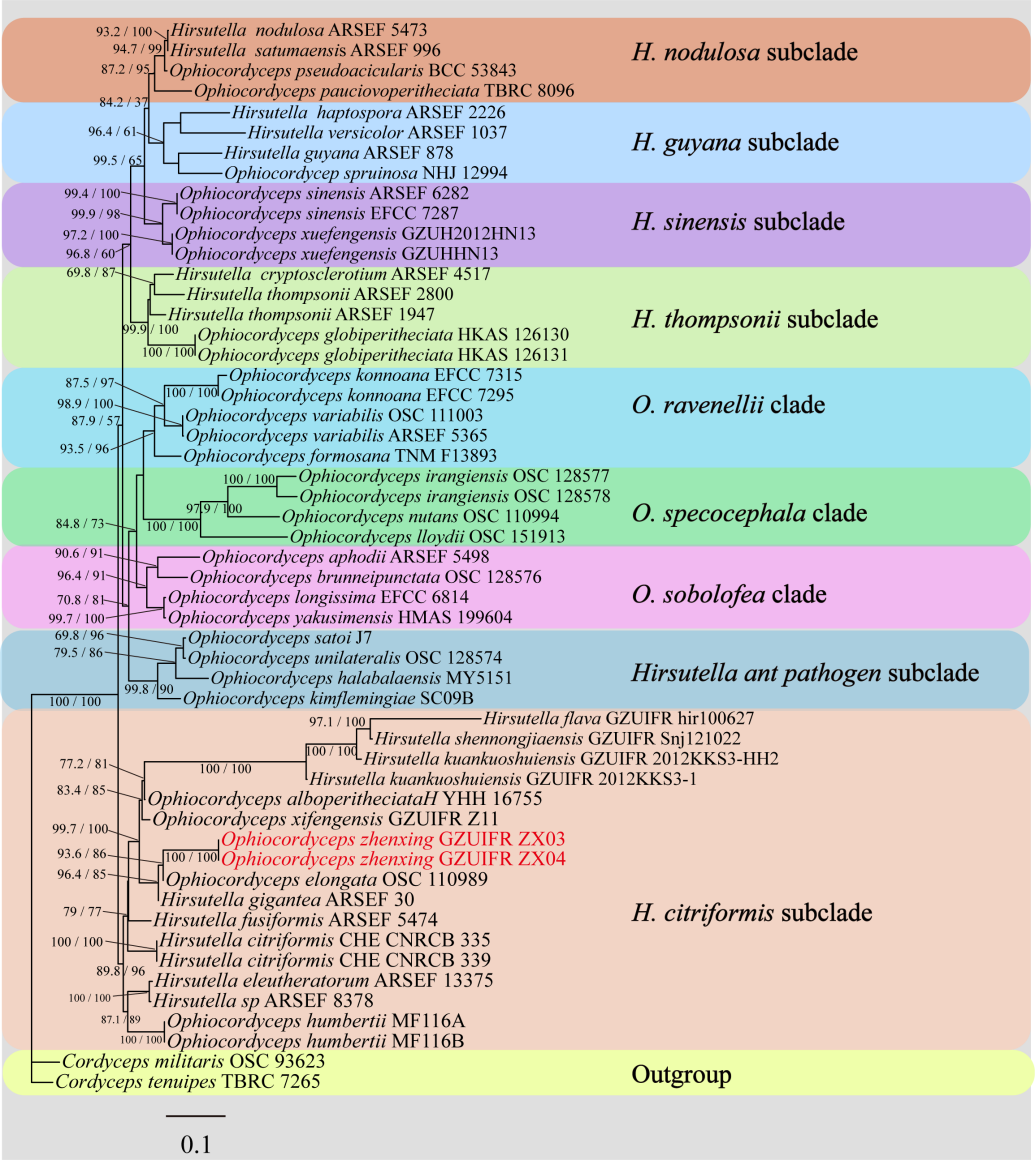
Phylogenetic tree based on the combined data set of nrSSU, nrLSU, *tef-1α*, *rpb1*, and ITS showing the relationship of *Ophiocordyceps zhenxingensis* with other Ophiocordyceps species. Values at the nodes before and after the backslash are BI posterior probabilities (BI-PP greater than 0.60) and ML bootstrap proportions (ML-BP greater than 50%), respectively. New species described in this paper are shown in bold.

Figure 1 The Bayesian Inference (BI) and the maximum likelihood (ML) phylogenetic trees were constructed with the combined data set of five loci, which was composed of 3,732 bp (460 bp for ITS, 905 bp for nrLSU, 977 bp for nrSSU, 829 bp for *tef-1α*, and 561 bp for *rpb1*), representing 61 taxa for revealing the phylogenetic relationships of *Ophiocordyceps*, and two species, *Cordyceps militaris* OSC 93623 and *Cordyceps tenuipes* TBRC7265, were designated as outgroup (Fig. 1; Table 1). *Ophiocordyceps* consisted of four statistically well-supported clades: *Hirsutella* clade, *O. sphecocephala* clade, *O. sobolifera* clade, and *O. ravenelii* clade. Notably, the *Hirsutella* clade was subdivided into six distinct subclades, namely *H. sinensis*, *H. nodulosa*, *H. thompsonii*, *H. guyana*, *H. citriformis*, and *Hirsutella ant pathogen* subclades. As revealed from phylogenetic analyses, our specimens collected in this study were placed in the *H. citriformis* subclade. Two samples (GZUIFR ZX03, GZUIFR ZX04), newly described as *Ophiocordyceps zhenxingensis*, were clustered closely with *H. gigantea*, *O. elongata*, *O. alboperitheciata*, and *O. xifengensis*.

**TABLE 1:** Specimen information and GenBank accession number for sequences used in this study^*a*^^,*b*^

Species	Voucher information	GenBank accession no.				
		ITS	nrLSU	rpb1	tef-1α	nrSSU
*Cordyceps militaris*	OSC 93623	JN049825	AY184966	DQ522377	DQ522332	AY184977
*Cordyceps tenuipes*	TBRC 7265	MF140741	MF140707	MF140776	-	-
*H. citriformis*	CHE-CNRCB 339	-	-	KY587214	KY587204	KY587217
*H. citriformis*	CHE-CNRCB 335	-	-	KY587213	KY587203	KY587216
*H. citriformis* (*Cixiidae*)	ARSEF 1035	KM652153	KM652105	KM652030	KM651989	KM652064
*H. citriformis* (*Psyllidae*)	ARSEF 2598	KM652155	KM652107	-	KM651991	-
*H. cryptosclerotium*	ARSEF 4517	KM652157	KM652109	KM652032	KM651992	KM652066
*H. eleutheratorum*	ARSEF 13375	-	-	MH057733	MH057732	MH057734
*H. flava*	GZUIFR-hir100627-1	KY415598	KY415599	KY945366	KY415601	-
*H. fusiformis*	ARSEF 5474	-	KM652110	KM652033	KM651993	KM652067
*H. gigantea*	ARSEF 30	-	JX566977	KM652034	JX566980	-
*H. guyana*	ARSEF 878	KM652158	KM652111	KM652035	KM651994	KM652068
*H. haptospora*	ARSEF 2226	KM652159	-	KM652036	KM651995	-
*H. kuankuoshuiensis*	GZUIFR 2012KKS3-1	KY415575	KY415582	KY945360	KY415590	-
*H. kuankuoshuiensis*	GZUIFR 2012KKS3-2	MF623038	MF623044	-	MF623048	-
*H. nodulosa*	ARSEF 5473	KM652165	KM652117	KM652040	KM652000	KM652074
*H. satumaensis*	ARSEF 996	KM652172	KM652125	KM652047	KM652008	KM652082
*H. shennongjiaensis*	GZUIFR-Snj121022	-	KY945357	KY945364	-	-
*H. thompsonii*	ARSEF 1947	KM652191	KM652146	-	KM652026	-
*H. thompsonii*	ARSEF 2800	KM652187	KM652142	KM652058	KM652023	KM652095
*H. versicolor*	ARSEF 1037	-	KM652150	KM652063	KM652029	KM652102
*Hirsutella* sp.	ARSEF 8378	-	KM652127	KM652049	KM652010	KM652084
*O. alboperitheciata*	YHH 16755	-	MT222278	MT222280	MT222279	-
*O. aphodii*	ARSEF 5498	-	DQ518755	-	DQ522323	DQ522541
*O. brunneipunctata*	OSC 128576	-	DQ518756	DQ522369	DQ522324	DQ522542
*O. elongata*	OSC 110989	-	EF468808	EF468856	EF468748	-
*O. elongata*	OSC 110989	-	EF468808	EF468856	EF468748	-
*O. formosana*	TNM F13893	-	-	KJ878988	KJ878956	KJ878908
*O. globiperitheciata*	HKAS 126130	OR015963	OR015968	OR119834	OR030532	OR082950
*O. globiperitheciata*	HKAS 126131	OR015964	OR015969	OR119835	OR030533	OR082951
*O. halabalaensis*	MY5151	GU723763	-	-	GU797110	KM655826
*O. humbertii*	MF116B	-	MK875536	MK863829	-	MK874748
*O. humbertii*	MF116A	-	MK875537	MK863828	-	MK874747
*O. irangiensis*	OSC 128578	JN049833	DQ518770	DQ522391	DQ522345	DQ522556
*O. irangiensis*	OSC 128577	JN049823	DQ518760	DQ522374	DQ522329	DQ522546
*O. kimflemingiae*	SC09B	-	KX713620	KX713724	KX713698	KX713631
*O. konnoana*	EFCC 7315	-	-	EF468861	EF468753	EF468959
*O. konnoana*	EFCC 7295	-	-	EF468862	-	EF468958
*O. lloydii*	OSC 151913	-	KJ878891	KJ879004	KJ878970	KJ878924
*O. longissima*	EFCC 6814	-	EF468817	EF468865	EF468757	-
*O. nujiangensis*	YFCC 8880	-	ON723381	ON868823	ON868820	ON723384
*O. nujiangensis*	YHH20041	-	ON723383	ON868825	ON868822	ON723385
*O. nutans*	OSC 110994	-	DQ518763	DQ522378	DQ522333	DQ522549
*O. sobolifera*	TNS F18521	-	KJ878898	KJ879013	KJ878979	KJ878933
*O. sobolifea*	NBRC 106967	AB968409	AB968422	-	AB968590	AB968395
*O. sphecocephala*	NBRC 101752	JN943351	JN941445	JN992430	AB968591	JN941696
*O. sphecocephala*	NBRC 101753	JN943350	JN941446	JN992429	AB968592	JN941695
*O. pruinosa*	NHJ 12994	-	EU369041	EU369036	EU369024	EU369106
*O. pseudoacicularis*	BCC 53843	-	MF614646	MF614661	MF614630	-
*O. pauciovoperitheciata*	TBRC 8096	-	MF614651	MF614634	MF614666	-
*O. ravenelii*	OSC 110995	-	DQ518764	DQ522379	DQ522334	DQ522550
*O. sinensis*	EFCC 7287	JN049854	EF468827	EF468874	EF468767	EF468971
*O. sinensis*	ARSEF 6282	KM652173	KM652126	KM652048	KM652009	KM652083
*O. satoi*	J7	-	KX713599	KX713711	KX713683	KX713653
*O. unilateralis*	OSC 128574	-	DQ518768	DQ522385	DQ522339	DQ522554
*O. variabilis*	OSC 111003	-	EF468839	EF468885	EF468779	EF468985
*O. variabilis*	ARSEF 5365	-	DQ518769	DQ522386	DQ522340	DQ522555
*O. xifengensis*	GZUIFR Z11	OQ947874	OQ948160	OR014499	OR014500	OQ948145
*O. xuefengensis*	GZUH2012HN13	KC631801	-	KC631797	KC631792	KC631787
*O. xuefengensis*	GZUHHN13	KC631804	-	KC631795	KC631790	KC631785
*O. yakusimensis*	HMAS 199604	-	KJ878902	KJ879018	-	KJ878938
***O. zhenxingensis***	**GZUIFR ZX03**	**PP464865**	**PP593601**	**PP506091**	**PP506092**	**PV400557**
***O. zhenxingensis***	**GZUIFR ZX04**	**PV400547**	**PQ159488**	**PQ164150**	**PQ164149**	**PV400563**

^*a*^ The bold represents the species and related information reported in this study.

^*b*^ "-" indicates that there is no corresponding information in the database.

However, the phylogenetic evidence indicated that these two samples formed a monophyletic clade in *Ophiocordyceps*, with high statistical support (BI-PP/ML-BS = 1/100). Therefore, the phylogenetic data supported the recognition of *O. zhenxingensi* as a distinct species in *Ophiocordyceps*.

### Morphology

**Fig 2:**
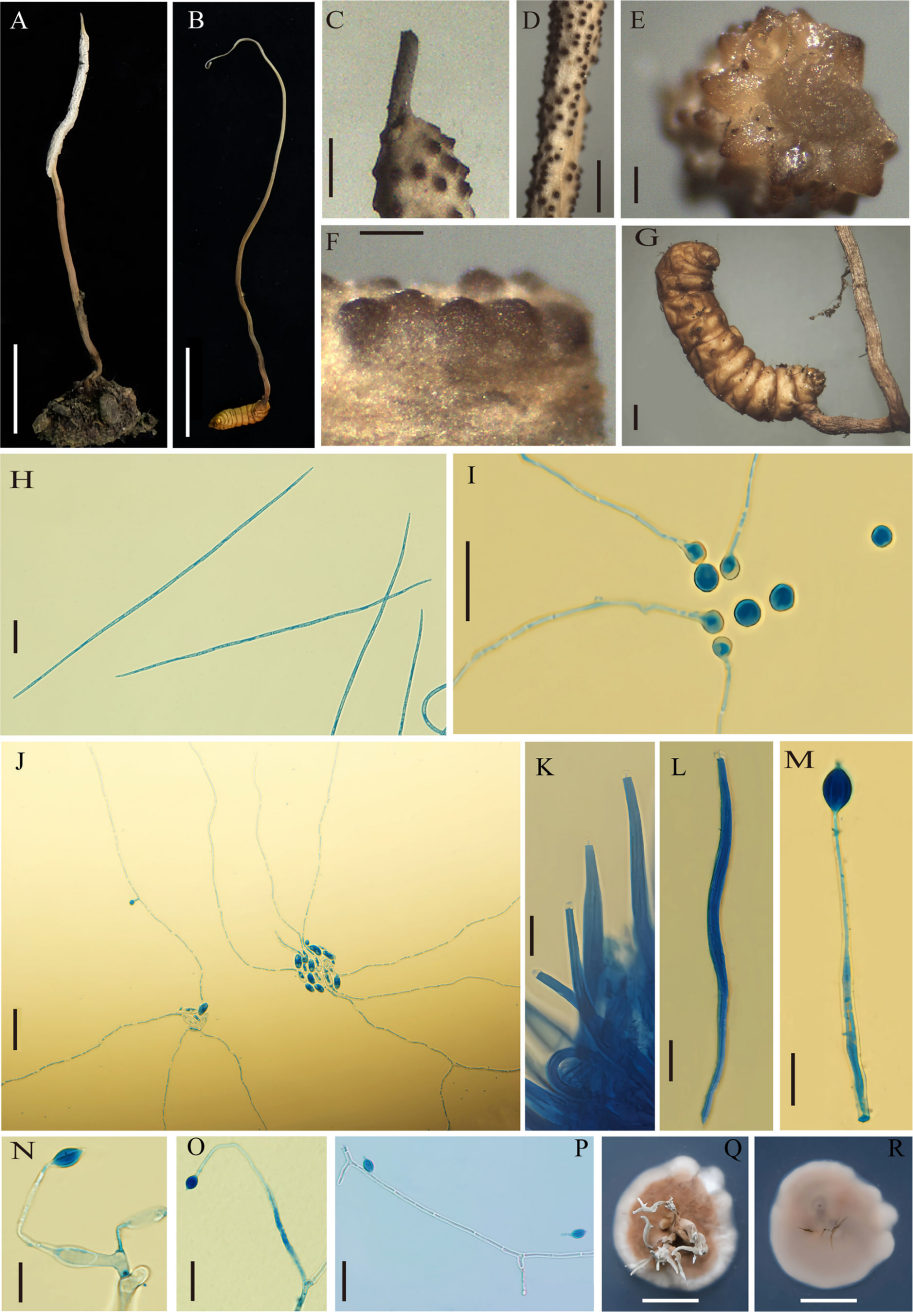
*Ophiocordyceps zhenxingensis* (**A**) sexual stromata of fungus arising from lepidopterous larvae, (**B**) asexual stromata of fungus arising from lepidopterous larvae, (**C**) fertile part with infertile tip, (**D through F**) perithecia arrangement, (**G**) host, (**H**) ascospores, (**I and J**) spore germination, (**K and L**) asci, (**M through P**) conidiogenous cells and conidia, (**Q and R**) colony on PDA. Scale bars: 1 cm (**A, B, Q, R**) ,0.1 mm (**C**), 0.2 mm (**D, F**), 1 mm (**E, G**), 20 µm (**H, J, K, L, O, P**), and 10 µm (**I, M, N**).

Figure 2 *Ophiocordyceps zhenxingensis*: H.L. Tian and X. Zou, sp. nov.

*Fungal name*: FN57203

*Etymology*: referring to the locality of the specimen collected, Zhenxing (Lat. “zhenxingensis”).

*Holotype*: Zhenxing Town, Xifeng County, Tieling City, Liaoning Province, China (42°6.38′N, 125°0.06′E), 8 July 2023 on Apidae (Hymenoptera) larvae (holotype, GZUIRF ZX03; ex-type, GZUIR ZX04).

*Description*: stromata mostly grew from the head of Apidae (Hymenoptera) larvae, a few from the tail and abdomen, solitary or multiple cylindrical, mostly erect, 3–6 cm long, woody, hard, fertile part off-white, infertile part light brown to brown, mostly with an infertile tip. Perithecia were inclined and semi-submerged, conical, the exposed part was dark brown, and the buried part was pale yellow, 0.29–0.35 × 0.21–0.25 mm in size. Asci were cylindrical with an apical cap, measuring 201.6–238.2 × 7.7–8.2 µm. Ejected ascospores were filiform and septate, measuring 201.6–232.7 × 2.2–3.2 µm. Ascospores were globose or ellipsoidal when they germinate.

*Culture characteristics*: colonies were 17–19 mm in diameter when cultured on potato dextrose agar (PDA) medium at 20°C for 30 days; the colony surface was creamy white around, brown inside, and light brown on the reverse side, whereas synnemata grew on the front side. Conidiogenous cells were present on single pedicels and pedicels of two types (A and B). The base of type A was enlarged (15.2–19.5 × 3.6–5.0 µm) and the neck gradually tapered (21.3–24.9 × 0.5–1.8 µm). The base of type B was not enlarged (95.2–104.5 × 1.5–2.6 µm). The conidia were fusiform, produced by the apical part of the hyphae (8.9–11.6 × 4.9–6.2 µm) with sporulating mucilage; hyphae were septate.

### Analysis of the microbial community composition and diversity

**Fig 3:**
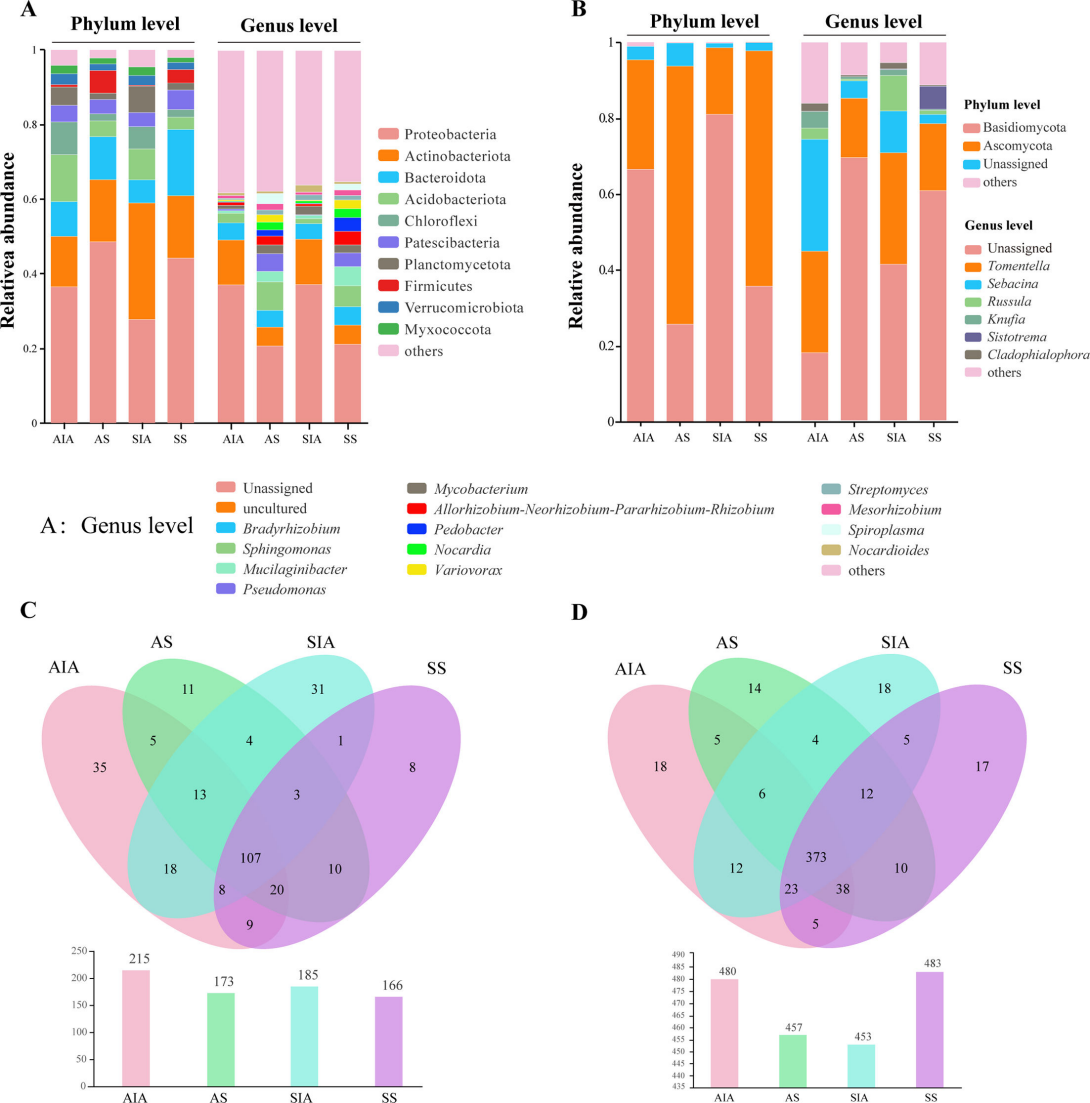
The microbial composition of *O. zhenxingensis.* (**A**) The composition of the bacterial community at the phylum and genus levels. (**B**) The composition of the fungal community at the phylum and genus levels. (**C**) Venn diagrams of fungi at the genus level. (**D**) Venn diagrams of bacteria at the genus level. “Uncultured” denotes that the taxonomic classification of the microorganism is based solely on sequence similarity, in the absence of an associated pure culture isolate. *A-N-P-R*: *Allorhizobium-Neorhizobium-Pararhizobium-Rhizobium*.

Within the bacterial community composition, there existed 10 phyla with a relative abundance of >1% in each group. Proteobacteria, Actinobacteriota, and Bacteroidota were the dominant phyla shared by each group (relative abundance >5%). Proteobacteria were predominant in all groups, constituting a higher percentage of sclerotium than host surface complex (36.48% in asexual host surface complex [AHSC] and 27.72% in sexual host surface complex [SHSC]; 48.54% in asexual sclerotium [AS] and 44.20% in sexual sclerotium [SS]). Furthermore, the AHSC and SHSC groups also shared two dominant phyla, namely Acidobacteriota and Chloroflexi. Planctomycetota was dominant only in SHSC, Firmicutes was dominant only in AS, and Patescibacteria was dominant only in SS (Fig. 3A). At the genus level, the bacterial community composition was similar between AHSC and SHSC, and between AS and SS, whereas there was a difference between sclerotium and host surface complex (Fig. 3A). Among the top 15 bacterial taxa in terms of abundance, except for unassigned and uncultured, the remaining 13 genera comprised 12.64% in AHSC, 36.34% in AS, 14.48% in SHSC, and 38.37% in SS (Fig. 3A). The relative abundance of *Bradyrhizobium* was higher in AHSC and SHSC, attaining 4.59% in the former and 4.19% in the latter. Meanwhile, the relative abundance of *Sphingomonas* was higher in both AS and SS, with 7.64% in the former and 5.60% in the latter.

At the phylum level, the fungal community consisted mainly of Basidiomycota and Ascomycota, except for an unassigned portion, and Ascomycota was the most dominant in AHSC (66.43%) and SHSC (80.94%), whereas only Ascomycota was most dominant in AS (68.00%) and SS (62.12%; Fig. 3B). At the genus level, six genera (*Tomentella*, *Sebacina*, *Russula*, *Knufia*, *Sistotrema*, and *Cladophialophora*) had a relative abundance of >1% and unassigned. In AHSC, unassigned (17.94%), *Tomentella* (26.81%), and *Sebacina* (29.57%) were the dominant taxa comprising the community; in SHSC, *Russula* (9.41%) was the dominant genus in addition to the above three sections. In AS and SS, the unassigned were the most dominant taxa constituting the community, with 69.50% in the former and 60.72% in the latter; *Tomentella* was the dominant genus in endomycorrhiza, with 15.70% in AS and 17.72% in SS (Fig. 3B).

Based on the original operational taxonomic unit (OTU) statistics, at the genus level, there were 107 fungal genera in the four sample groups; 35, 31, 11, and 8 genera were unique to AHSC, SHSC, AS, and SS, respectively, and the genera contained in each group were 215, 185, 173, and 166, respectively (Fig. 3C). There were 373 bacterial genera; the genera specific to AHSC, SHSC, AS, and SS were 18, 18, 14, and 17, respectively, and the genera contained in each group were 480, 453, 457, and 483, respectively (Fig. 3D). Overall, in the host surface complex, the number of fungal and bacterial OTUs was higher in the asexual than the sexual stages; in the sclerotium, the number of fungal OTUs was higher in the asexual than the sexual stages, whereas the number of bacterial OTUs was lower than in the sexual stage.

**Fig 4:**
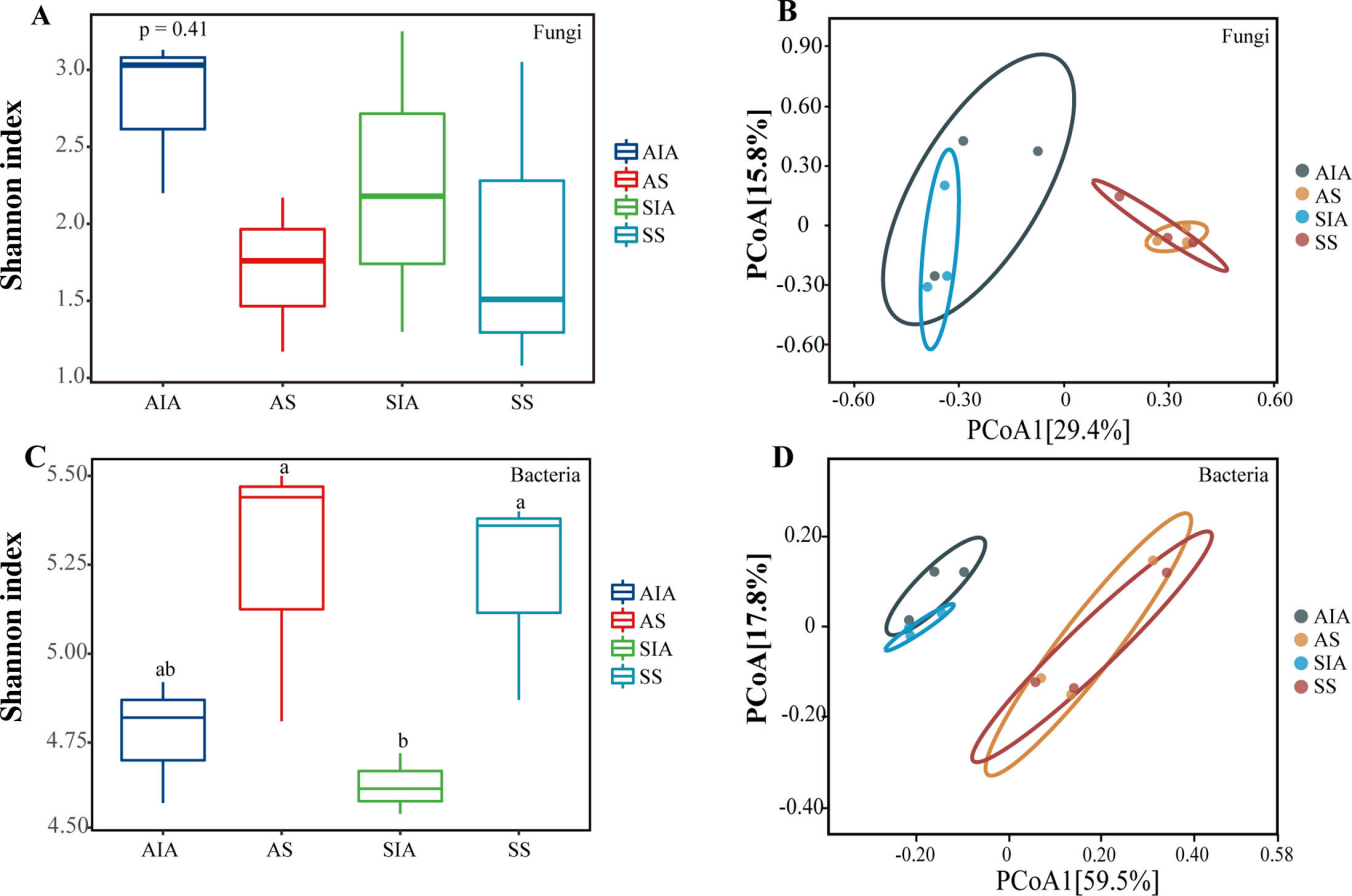
Species diversity. (**A**) The alpha diversity of fungi. (**B**) The beta diversity of fungi. (**C**) The alpha diversity of bacteria. (**D**) The beta diversity of bacteria. Different lowercase letters represent statistically significant differences (*P* < 0.05).

Among fungal communities, the Shannon index showed that AHSC had the highest α-diversity, followed by SHSC, SS, and AS, i.e., host surface complex had higher diversity than sclerotium (Fig. 4A). In contrast, in the bacterial community, AS had the highest α-diversity, followed by SS, AHSC, and SHSC. There was a significant difference between SHSC and AS and SS (*P* < 0.05). Overall, the bacterial α-diversity was higher in sclerotium than in host surface complex (Fig. 4C). Principal coordinate analysis (PCoA) showed that the fungal and bacterial communities in host surface complex and sclerotium showed clear differentiation and were more similar in microbial composition between the asexual and sexual stages (Fig. 4B and D), where fungi were more clustered in the sclerotium (Fig. 4B) and bacteria in host surface complex (Fig. 4D).

### Characterization of the microbial network structure

**Fig 5:**
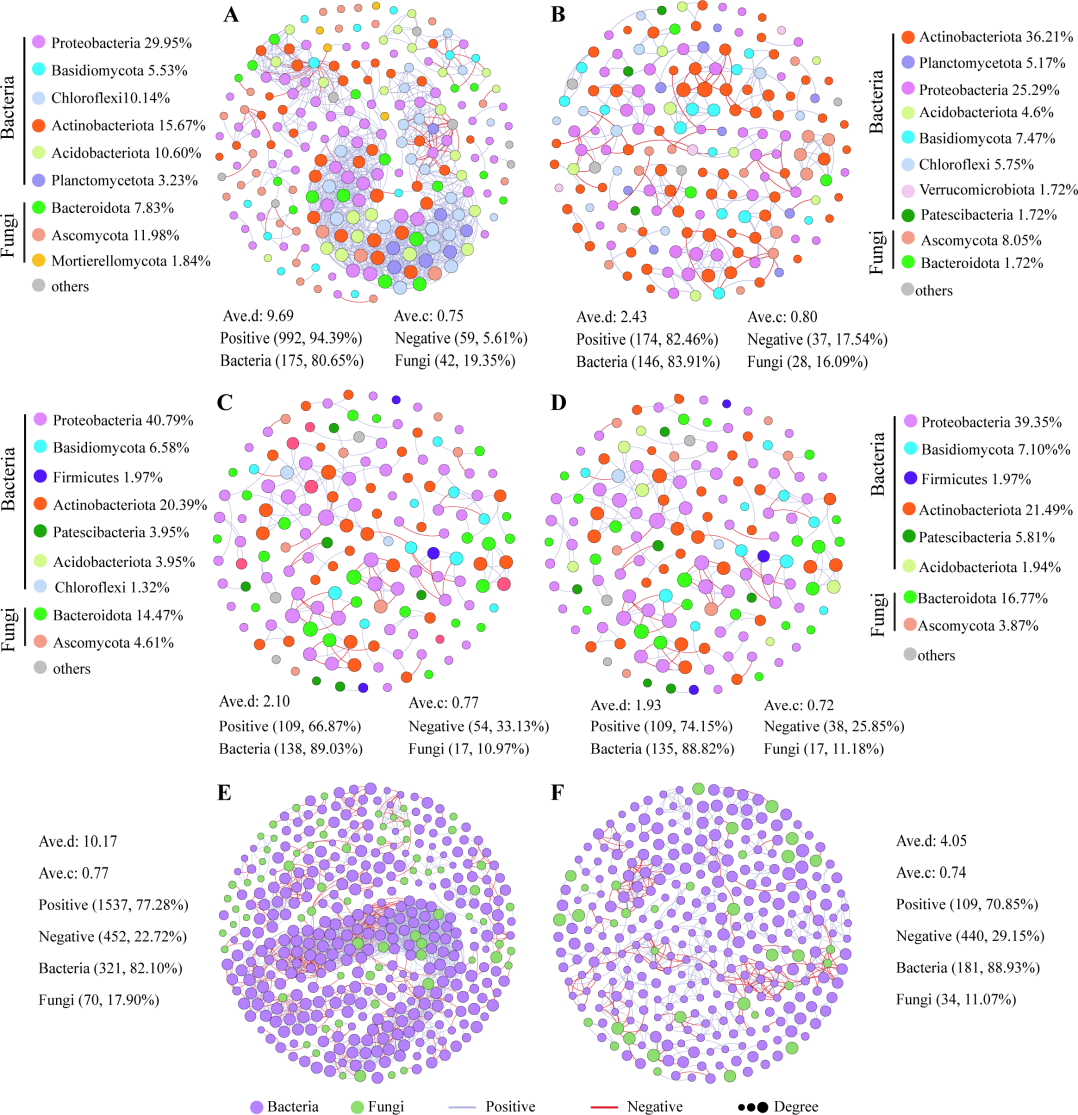
Microbial network structure characterization (OTUs greater than 1% of sequence sum): (**A**) asexual host surface complex, (**B**) sexual host surface complex, (**C**) asexual sclerotium, (**D**) sexual sclerotium, (**E**) host surface complex, and (**F**) sclerotium.

**Fig 6:**
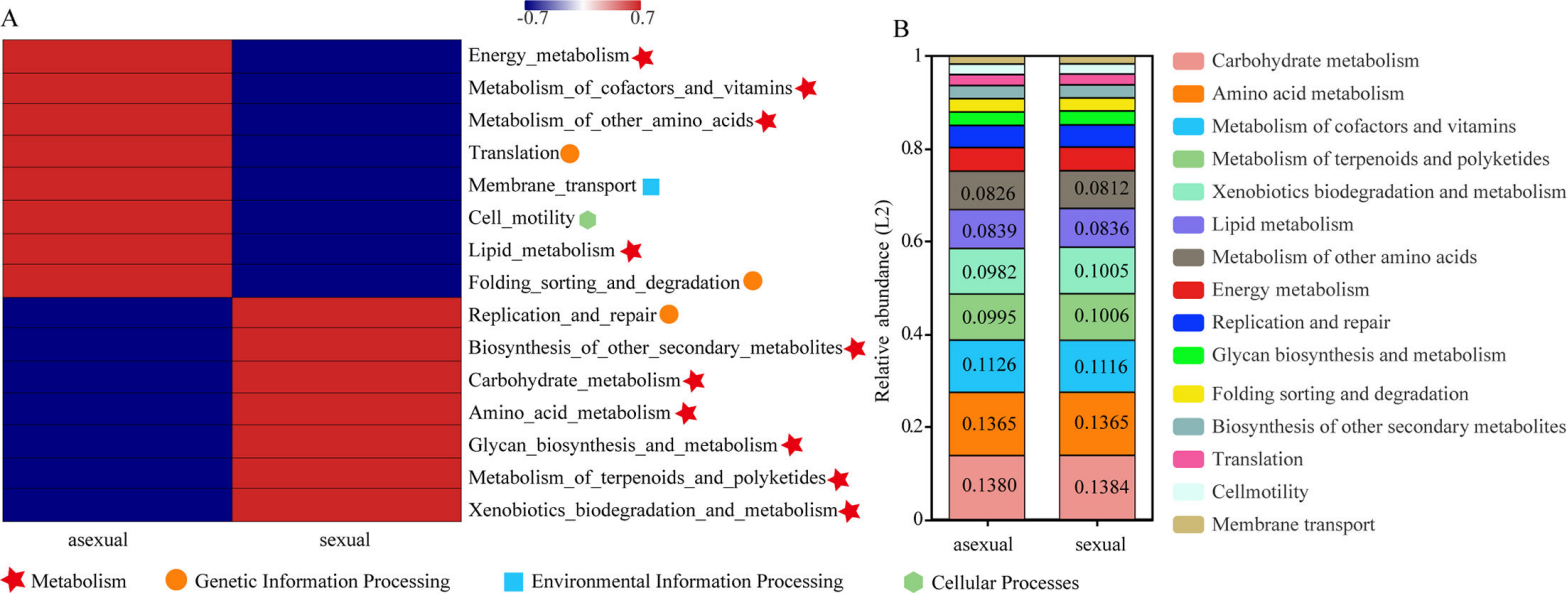
KEGG pathway composition of bacteria: (**A**) pathway clustering at the secondary classification level, (**B**) pathway abundance composition at the secondary classification level. Different symbols in the figure represent different KEGG pathways at the primary classification level.

The microbial co-occurrence network of AHSC was the most complex and aggregated (217 nodes and 1,051 edges; Fig. 5A), followed by SHSC (174 nodes and 211 edges; Fig. 5B), SS (155 nodes and 163 edges; Fig. 5C), and AS (152 nodes and 147 edges; Fig. 5D). The microbial interactions within *O. zhenxingensis* were predominantly positive correlations (66.87–94.39%; Fig. 6A through D). The positive correlations were more pronounced in host surface complex than in sclerotium (Fig. 5E and F), and the highest positive correlations were detected in the AHSC (94.39%; Fig. 5A). The average network connectivity in AHSC (9.69%) was higher than in the other three groups, whereas the average clustering coefficient was the highest in SHSC (Fig. 6A through D). Results also indicated that bacteria dominated the samples in all groups (Fig. 5), and the microbial diversity was higher in the host surface complex than in sclerotium (Fig. 5E and F).

### Functional forecasting analysis

The composition of the Kyoto Encyclopedia of Genes and Genomes (KEGG) pathways revealed that the top 15 pathways were predominantly metabolic. The abundance related to carbohydrate metabolism holds the top position, followed by amino acid metabolism, cofactor and vitamin metabolism, etc. (Fig. 6A). There was no significant difference in the composition of the metabolic pathways and associated bacterial abundance between the two different reproductive stages (Fig. 6B). However, in terms of pathway clustering, there were significant differences in the relationships between bacteria and related pathways at different reproductive stages, with energy metabolism, metabolism of cofactors and vitamins, metabolism of other amino acids, lipid metabolism, translation, membrane transport, cell motility, and folding sorting and degradation dominating at the asexual reproduction stage. In contrast, the sexual reproduction stage was dominated by xenobiotics biodegradation and metabolism, Metabolism of terpenoids and polyketide, metabolism of terpenoids and polyketides, amino acid metabolism, carbohydrate metabolism, biosynthesis of other secondary metabolites, and replication and repair (Fig. 6B).

## DISCUSSION

Phylogenetic trees constructed using BI and ML methods showed that *O. zhenxingensis* was closely related to *H. gigantea* and *O. elongata* with strong support. According to the law of botanical nomenclature (one fungus = one name), it is required that the two taxonomic systems, sexual and asexual, are harmonized (25). Simmons et al. (23) also confirmed that *H. gigantea* is an asexual stage of *O. elongata* and supported the combination of the two. The host of *O. elongata* was lepidopteran pupae. The synnemata exhibit numerous lateral branches in the middle to upper portions, with the main synnemata appearing dark brown and the branches tinged gray. The phialides possess distinctly swollen basal regions that are oblong to subellipsoid, measuring 10–13 × 5–6.4 µm. The conidia are subfusiform to orange-segment-shaped, 6–9 × 2–3.5 µm (26). In contrast, *O. zhenxingensis* parasitizes hymenopteran larvae. The synnemata are unbranched, with the fertile portion appearing whitish-gray and the sterile part pale to dark brown. Two types of phialides are observed: one with a distinctly swollen base and the other tubular without basal swelling. The conidial morphology resembles that of *O. elongata* but differs in size; notably, the conidia of *O. zhenxingensis* (8.9–11.6 × 4.9–6.2 µm) are larger than those of *O. elongata*.

Furthermore, phylogenetic analyses placed *O. zhenxingensis* in a close relationship with *O. alboperitheciata* and *O. xifengensis*, with each species forming a distinct, well-supported clade. Morphologically, *O. zhenxingensis* differs from *O. alboperitheciata* in several key aspects: perithecial arrangement and dimensions (290–350 × 210–250 µm in *O. zhenxingensis*, 408–549 × 233–321 µm in *O. alboperitheciata*), ascus size (201.6–238.2 × 7.7–8.2 μm, 144–246 × 3.5–4.7 µm, respectively), and colony morphology on PDA; *O. alboperitheciata* produces rugose colonies lacking synnemata, whereas *O. zhenxingensis* forms flat colonies with distinct synnemata (5). The most striking distinctions between *O. zhenxingensis* and *O. xifengensis* lie in host specificity (Hymenoptera vs Lepidoptera) and phialide neck morphology. *O. xifengensis* exhibits conspicuously tapered and helically coiled phialide necks, whereas those of *O. zhenxingensis* lack these features (6). A morphological comparison of *O. zhenxingensis* and its allies is provided in Table 2.

**TABLE 2:** A morphological comparison of *Ophiocordyceps zhenxingensis* and its allies^*a*^

Species	Host	Stromata	Ascomata	Asci	Ascospores	Conidiogenous cells	Conidia	Reference
*H. citriformis*	Adult of Fulgoridae (Hemiptera)	Flexible, simple or branched, brown	-	-	-	Sporophores simple, sessile or subsessile, with rather short, delicate sterigmata 20–30 µm	Fusiform, 5.5–8.5 × 1.5–18 µm	(27)
*H. kuankuoshuiensis*	Larva Lepidoptera	Solitary, brown, up to 86.0 mm	-	-	-	Borne perpendicular slender columnar a long and narrow neck 30–45 × 1–3 µm long	narrow fusiform single—or double—enveloped in a hyaline mucus 9.9–12.6 × 2.7–4.5 µm	(28)
*H. shennongjiaensis*	Earwig Dermaptera	Synnemata, cylindrical, 60.0 × 1.0–2.0 mm, brown	-	-	-	Solitary, phialides cylindrical or awlike, 14.4–26.1 or 6.3–14.4 µm	Conidia hyaline, sausage-shaped, single or double from the apex of the neck	(29)
*O. alboperitheciata*	Larva of Noctuidae (Lepidoptera)	Multiple, unbranched, light brown to dark brown, up to 54–65 mm, clavate fertile part, white to light brown	Superficial, scattered or crowded, 0.41–0.55 × 0.23–0.32 mm, nearly ovoid, white nearly light brown	Cylindrical, 8-spores, 144–246 × 3.5–4.7 µm, with a hemispheric apical cap, 3.2–4.2 × 2.3–2.5 µm	Cylindric, multiseptate, 0.5–0.6 µm diameter, with septa 1.1–1.3 µm apart. Part-spores were not seen	-	-	(30)
*O. elongata*	Pupae and larvae of *Apalela americana* (Lepidoptera)	Flexuose, numerous lateral branches, 110 mm long, pale brown	Immersed, scattered or crowded, ovato conoid, 0.5 × 0.3 mm, apex subacute, wall yellow by transmitted light	The asci are 220 µm long, 8 mm diameter	Cylindric, 2 µm diameter, with septa 4–12 µm apart. Part-spores were not seen	Distinctly swollen basal regions, oblong to subellipsoid	Subfusiform to orange-segment-shaped, 6–9 × 2–3.5 µm	()(1, 26)
*O. xifengensis*	Pupae Lepidoptera	Solitary, simple, single dark brown to yellow, up to 90.0 mm long	Inclined and buried, conical, yellow 0.38–0.43 × 0.16–0.19 mm	Cylindrical, 164.8–178.9 × 7.4–10.2 µm, with an orange petal apical cap, 6.36–10.7 × 5.14–7.46 µm	Hyaline, filiform, multi-septate	Solitary, columnar, base not obviously expanded and expanded at the base, curved in a spiral or wavy shape, 16.0–35.7 × 1.6–4.2 or 11.4–18.1 × 1.0–3.7 µm	Hyaline, lemon or long ellipsoidal, 8.0–10.7 × 3.8–4.8 µm	(6)
*O. zhenxingensis*	Larvae of Hymenoptera	Solitary or multiple, 30–60 mm long, woody, hard, fertile part off-white, infertile part light brown to brown	Inclined and buried, the exposed part of the ascus is dark brown, and the buried part is pale yellow, 0.29–0.35 × 0.21–0.25 mm	Cylindrical, 201.6–221.2 × 7.7–8.2 µm	Filiform, septate, whole, 201.6–232.7 × 2.2–3.8 µm	The base of type A was enlarged, 15.2–19.5 × 3.6–5.0 µm, and the neck gradually tapered, 21.3–24.9 × 0.5–1.8 µm. The base of type B is not enlarged, 95.2–104.5 × 1.5–2.6 µm	Fusiform, 8.9–11.6 × 4.9–6.2 µm	This study

^*a*^ "-" indicates that there is no corresponding information in the database.

Collectively, both phylogenetic analyses and morphological characteristics demonstrate significant distinctions between *O. zhenxingensis* and its closely related species. These robust lines of evidence strongly support its recognition as a novel taxonomic entity.

Furthermore, with regard to microbial composition, Proteobacteria, Actinobacteriota, and Bacteroidota (bacteria) were dominant (relative abundance, >5%) and common to all groups, similar to those of *O. sinensis* (15). Conversely, Bacteroidota was not a dominant phylum in *C. militaris* (31). Proteobacteria were more predominant in sclerotium than in the host surface complex. Similarly, bacteria from the above two phyla were also dominant in the soil on the surface of *O. sinensis* and *C. militaris* membranes (15, 31). At the genus level, the abundance of *Bradyrhizobium* was higher in bacterial membranes, also similar to that of *O. sinensis* (15) and *C. militaris* (31). *Bradyrhizobium* is an extremely important group of commensal bacteria, which often form rhizomes in symbiosis with leguminous plants, thereby promoting nitrogen fixation in plants (32, 33). The KEGG metabolic pathway showed that the abundance of bacteria associated with nitrogen metabolism was also higher in the host surface complex. It can be inferred that *Bradyrhizobium* may play an important role in *O. zhenxingensis* and participate in nitrogen metabolism.

Basidiomycota and Ascomycota were the principal dominant fungal groups, also commonly encountered as dominant microbial groups in other studies related to *Ophiocordyceps* (15, 31, 34). Basidiomycota was highly abundant in the insect body appendages, whereas Ascomycota was abundant in endospores. In terms of the abundance of dominant groups, *O.·zhenxingensis* was most similar to *O.·highlandensis* rather than *C. militaris* (31). The dynamics and diversity of the fungal community regulate the relationship between fungi and insect larvae, influencing the growth and development of *Ophiocordyceps* (17, 35). This study discovered that *Tomentella*, *Sebacina*, and *Russula*, abundant in host surface complex closer to the soil and plant roots, belong to mycorrhizal fungi. Mycorrhizal fungi modify the microbial environment and community around the roots, increase the number of microbial species with antagonistic effects on pathogens in the soil, and inhibit the growth and development of pathogens, thereby facilitating the formation of *Ophiocordyceps* (36).

This study observed that the microbial co-occurrence network of *O. zhenxingensis* exhibits an increased number of nodes and more intricate relationships during the asexual stage. In a study on *Apostichopus japonicus*, it was discovered that energy metabolism and lipid metabolism-related metabolic pathways were upregulated during the sexual gland development stage, facilitating the formation of sexual glands (37). Compared to this study, the energy and lipid metabolism of the asexual stage of *Ophiocordyceps* were higher than those of the sexual stage (Fig. 6A); hence, these two functional pathways might play a crucial role in the development of the asexual stage of *Ophiocordyceps*. Sexual reproduction can increase genetic diversity, enabling *Ophiocordyceps* to better adapt to environmental changes. In this study, carbohydrate metabolism was higher in the sexual stage than in the asexual stage, and the possible reason for this might be that carbohydrate metabolism can modify microbial competition and enrich functional groups in the microbial community (38), allowing the sexual stage of *Ophiocordyceps* to better adapt to environmental changes.

In *Ophiocordyceps*, the asexual phase is characterized by rapid hyphal expansion and nutrient acquisition, while the sexual phase involves the formation of reproductive structures such as perithecia and ascospores, marking the completion of the life cycle (39, 40). Therefore, we speculate that the observed results in this study—higher microbial diversity, stronger positive network correlations, and more enriched KEGG pathways during the asexual phase—may be attributed to the fact that most asexual individuals are in an immature state, requiring complex life activities to sustain their growth. For instance, carbohydrate metabolism provides energy and metabolic precursors to support hyphal growth and sporulation; membrane transport pathways enable fungi to absorb nutrients, facilitating hyphal growth and sporulation; and Lipid metabolism synthesizes the lipids necessary for cell membranes and spore walls (41–43). In contrast, the enhanced secondary metabolism during the sexual phase is due to the synthesis of pigments and signaling molecules required for the formation of sexual spores (44).

### Conclusions

This study identified a novel species of the *H. citriformis* subclade line fungus from Liaoning Province, China, which was designated as *O. zhenxingensis*. This study found that *O. zhenxingensis* was closely related to *O. elongata* in terms of systematic evolution. The microbial composition of *O. zhenxingensis* was characterized by a higher diversity of microorganisms during the asexual stage and more positive correlations among microorganisms. Its metabolic pathways are also more abundant.

## MATERIALS AND METHODS

### Specimen collection and isolation

In July 2022 and July 2023, 80 asexual (Fig. S1) and 50 sexual (Fig. S2) specimens parasitizing (Hymenoptera) larvae were collected in Zhenxing Town, Xifeng County, Tieling City, Liaoning Province, China. The samples were collected from soil, and the specimens from the sexual and asexual stages were placed separately in sterile plastic containers and transported to the laboratory. The fresh specimens were observed for host type and photographed in their entirety using a Nikon digital camera (D7500). The strains were isolated using the tissue isolating method (34, 45) and cultured on a PDA medium supplemented with 70 mg/mL benzylpenicillin and 50 mg/mL streptomycin at a constant temperature of 20°C for 30 days. The specimen and its isolated strains GZUIFR ZX03 (from the sexual stage) and GZUIFR ZX04 (from the asexual stage) were deposited in the Institute of Fungal Resources of Guizhou University (GZUIFR), Guiyang, China.

### Observation of morphological characteristics

Referring to the method of Luangsa-ard et al. (46), the perithecia arrangement was observed under a stereomicroscope (Leica S9I, Germany); ascomycete shells were picked and fixed with lactic acid cotton wool orchid; and the morphology and size of perithecia, asci, ascospores, and apical caps were observed and measured. The morphology of the cultured colonies was photographed with a Nikon digital camera (D7500), and the spore-producing structures and conidia of the asexual stage were observed under a light microscope (OM, Axio Imager 2 Pol, Zeiss, Germany).

### DNA extraction, polymerase chain reaction (PCR) amplification, and sequencing

DNA was extracted from colonies (GZUIFR ZX03 and GZUIFR ZX04) on a PDA medium using a modified CTAB (Hexadecyl trimethyl ammonium Bromide) method (35, 47). Four nuclear genes were amplified using the recovered DNA, including the intranuclear ribosomal transcribed spacer region (ITS), the large subunit of rDNA (LSU), the translation elongation factor-1α (tef-1α), and the largest subunit sequences of RNA polymerase II (rpb1). The primers and PCR amplification procedures used in this study were referenced from Wang et al. (48) and Araújo et al. (49). The PCR products were detected by 1% agarose gel electrophoresis and subsequently sent to Prime Biotech for purification and sequencing.

### Phylogenetic analysis

The initial sequence peak maps were examined using Chromas version 2.6.5. Accurate, nonoverlapping sequences were selected for forward and reverse sequence splicing using DNAMAN 9.0.1.116 (Lynnon Biosoft, USA). To check for unambiguous nucleotides, the spliced sequences were imported into BioEdit version 7.0.9. Subsequently, the newly generated sequences were aligned with other sequences obtained from GenBank using MAFFT version 7 (50). Phylogenetic analyses were performed for each locus, followed by combinatorial analyses using tandem data sets of multiple loci (ITS, LSU, tef-1α, and rpb1). The concatenated sequences of ITS, LSU, tef-1α, and rpb1 were obtained using PhyloSuite version 1.2.1 (16, 51) under the “concatenate sequence” module. In the alignment, “?” indicates missing sequence data in the alignment. ModelFinder2 was selected as the best model for nucleotide substitution (50). The data sets were analyzed using the ML and BI methods. Phylogenetic trees were inferred using IQ-TREE (52, 53) using an edge-link partitioning model and 10,000 replicates of nonparametric bootstrapping. MrBayes (54) was used to perform the Bayesian phylogenetic analysis, and Markov chain Monte Carlo was run for 5 million generations to assess the convergence of the analysis.

In the phylogenetic analysis, gene sequences from 44 taxa belonging to *Hirsutella* and *Ophiocordyceps* and their exogenous taxa such as *C. tenuipes* (Peck) Kepler, B. Shrestha, Spatafora, and *C. militaris* (L.) Fr. were retrieved from GenBank and merged with the sequences in this study.

### Genomic DNA extraction, PCR amplification, and high-throughput sequencing

**Fig 7:**
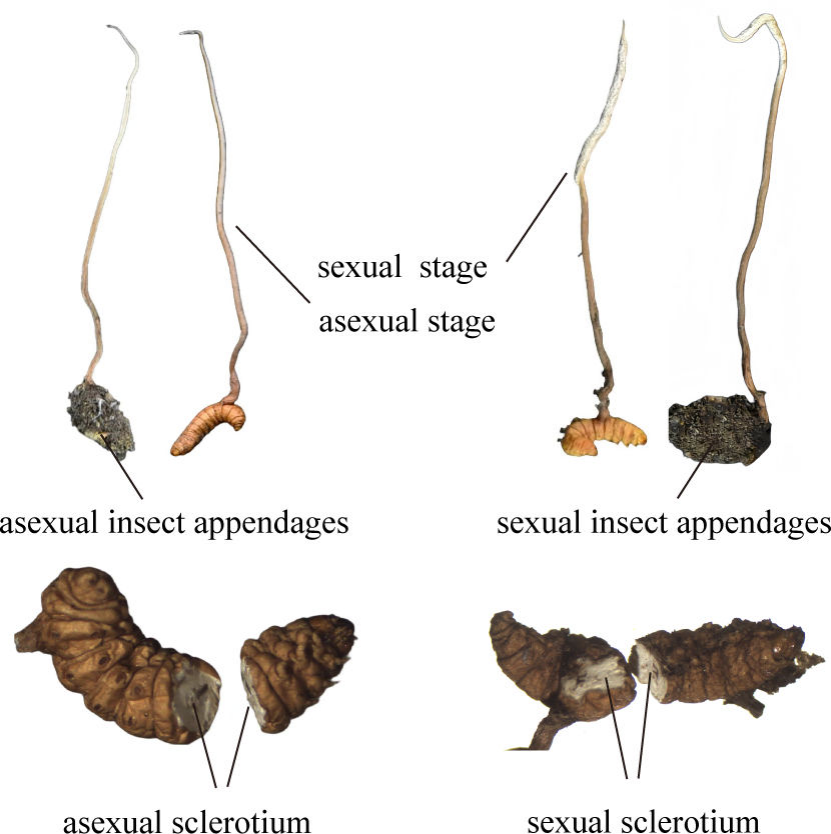
Schematic diagram of the structure of *O. zhenxingensis*. The left portion of the figure illustrates the phenotypic characteristics associated with the asexual stage, whereas the right portion depicts the characteristics of the sexual stage. The perithecia serve as the critical criterion for differentiating between these two stages.

Genomic DNA from AHSC, AS, SHSC, and SS of *O. zhenxingensis* (Fig. 7) was extracted using a Bacterial DNA Extraction Kit (Findrop) and tested for purity and concentration of DNA using Thermo NanoDrop One. Genomic DNA was used as a template for PCR amplification using specific primers with barcode and TaKaRa Premix Taq version 2.0 (TaKaRa Biotechnology Co., Dalian, China) according to the selection of the sequencing regions. The V3-V4 region (338F/806R) of the bacterial 16S rRNA gene and the ITS1-ITS2 region of the fungal 18S rRNA gene were targeted, and library construction was performed according to the standard NEBNext Ultra II DNA Library Prep Kit for Illumina (New England Biolabs, USA).

### Library construction

The constructed amplicon libraries were subjected to PE250 sequencing using the Illumina Nova 6000 platform (Guangdong Magigene Biotechnology Co., Ltd., Guangzhou, China). After sequencing, paired-end raw reads data filtering, paired-end clean reads splicing, and raw tag sequences for quality filtering were performed. After excluding chimeras to obtain representative OTU sequences, OTU sequences with at least 97% similarity were clustered in UPARSE (55). The obtained sequences were compared to the Silva 16S rRNA database (version 138), and the RDP classifier was used for species taxonomic annotation (56). Raw data were uploaded to the National Center for Biotechnology Information database under accession number PRJNA1085539 for fungi and PRJNA1085533 for bacteria.

### Data analysis

#### Species community analysis

α-Diversity analysis was based on the OTU abundance table using usearch-alpha-div (V10; http://www.drive5.com/usearch/) for the calculation of the α-diversity index and usearch-alpha-div-rare (V10; http://www.drive5.com/usearch/) for the dilution curve calculation of the diversity index. The Kruskal-Wallis rank-sum test was chosen for the analysis of differences between groups of the α-diversity index using R software.

### PCoA (default parameters)

Based on the OTU abundance table using the vegan package of R software based on the Bray-Curtis distance algorithm.

### Covariance network analysis

Based on the OTU abundance table, the core microbial (>1% abundance) species of the sexual and asexual stages were analyzed by co-occurrence network analysis using R software and mapped using Gephi 0.9.2.

### Prediction of KEGG function

The OTU abundance table was normalized by PICRUSt to remove the effect of the copy number of the 16S marker gene in the species genome. Through the greengene corresponding to each OTU, the comparison was made to the KEGG database. Based on the information from the comparison to the KEGG database, the pathway information was obtained. Based on the abundance of OTUs, the functional categories calculated the abundance of each sample at different categorization levels, respectively, and proceeded to the subsequent analysis.

## Data Availability

The raw sequence data have been deposited in the SRA database under accession number PRJNA1085539 for fungi and PRJNA1085533 for bacteria.

## SUPPLEMENTAL MATERIAL

Supplemental material spectrum.02159-24-s0001.docx Fig. S1 to S4.

ASM does not own the copyrights to Supplemental Material that may be linked to, or accessed through, an article. The authors have granted ASM a non-exclusive, world-wide license to publish the Supplemental Material files. Please contact the corresponding author directly for reuse.
