# Supplementary material for: Ophiocordyceps zhenxingensis sp. nov. and its microbiota during sexual and asexual stages in nature
Source: Microbiol Spectr. 2025 Jul 11;13(8):e02159-24. doi: 10.1128/spectrum.02159-24 (PMC12323668; doi:10.1128/spectrum.02159-24)
Supplement: Supplemental material — Fig. S1 to S4. [file spectrum.02159-24-s0001.docx]

**Supplementary materials**
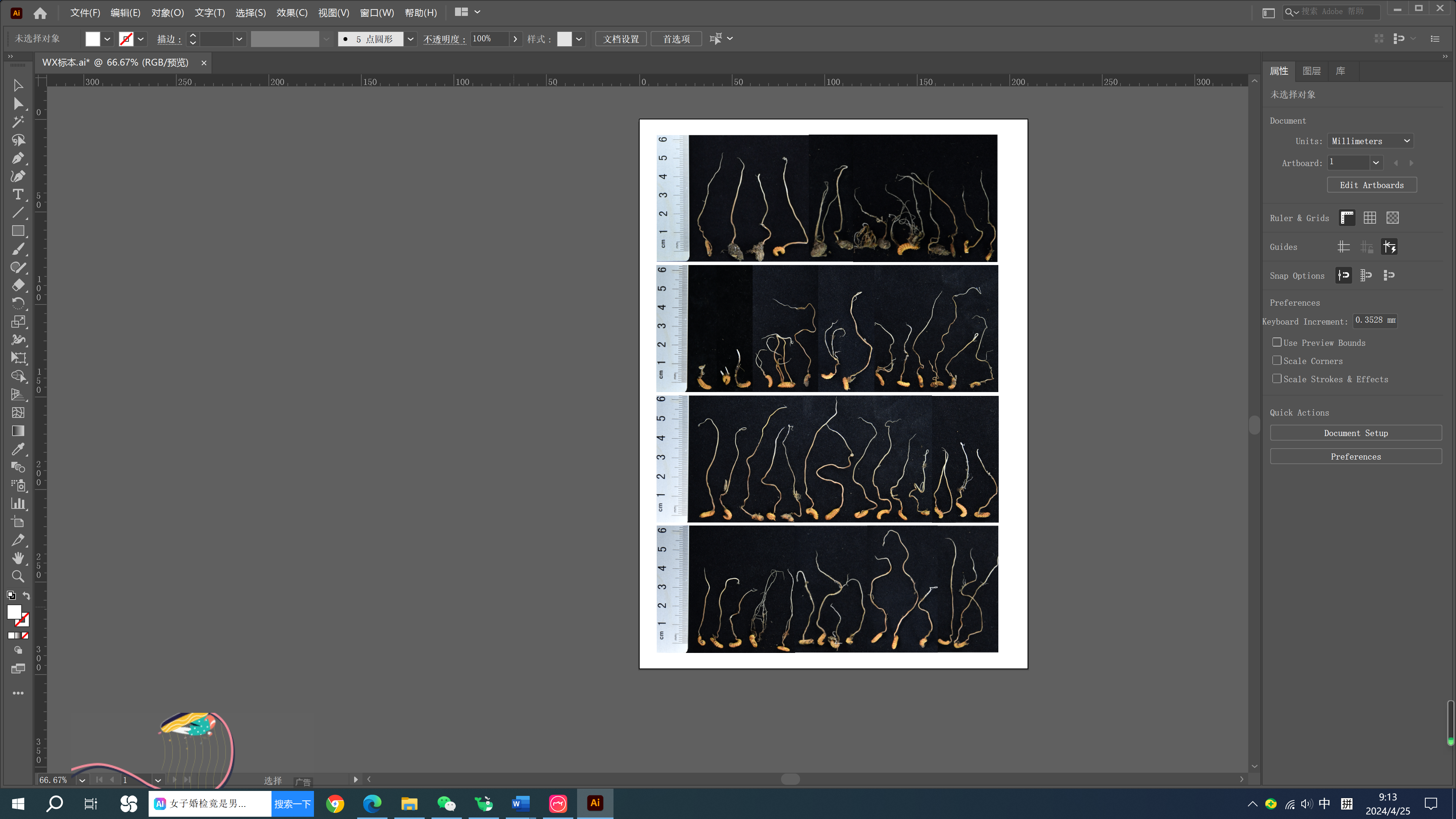


**Fig S1.** Wild asexual specimens. **Note**: *Ophiocordyceps zhenxingensis* specimens of the asexual stage (with some specimens not photographed), all of which were collected from forests in Zhenxing Town, Xifeng County, Tieling City, Liaoning Province, China.
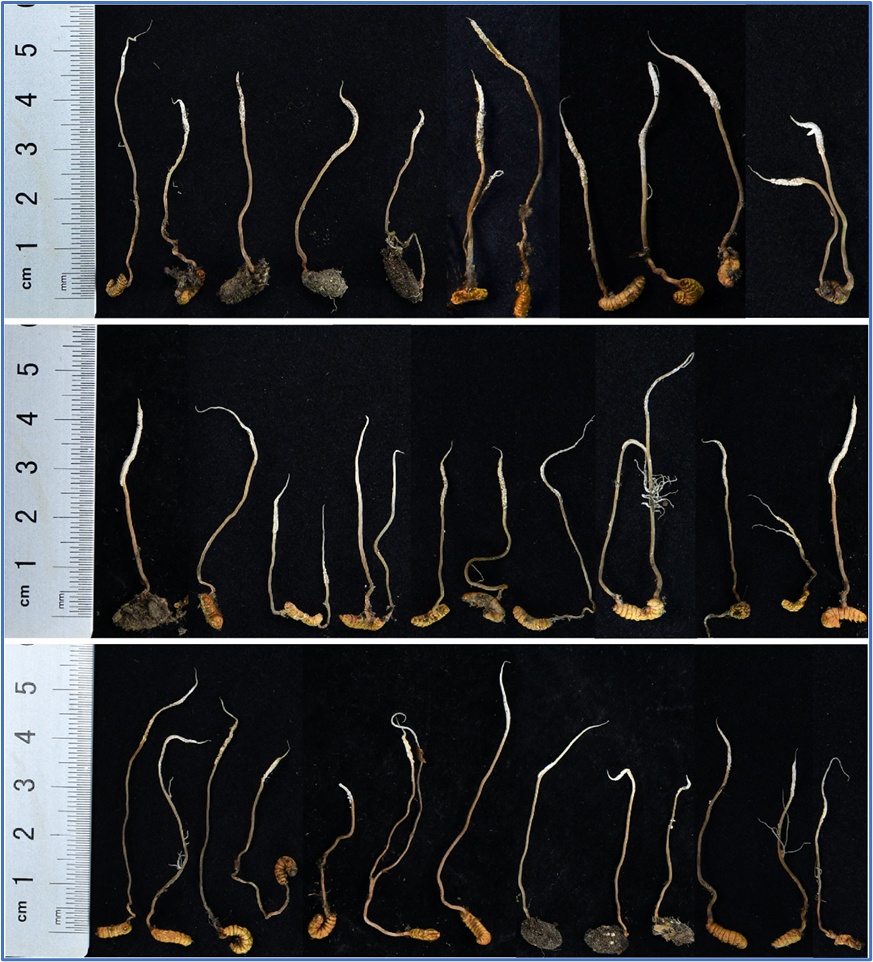


**Fig S2.** Sexual wild specimens. **Note**: *Ophiocordyceps zhenxingensis* specimens of the sexual stage (with some not photographed), all of which were collected from forests in Xifeng County, Tieling City, Liaoning Province.

**
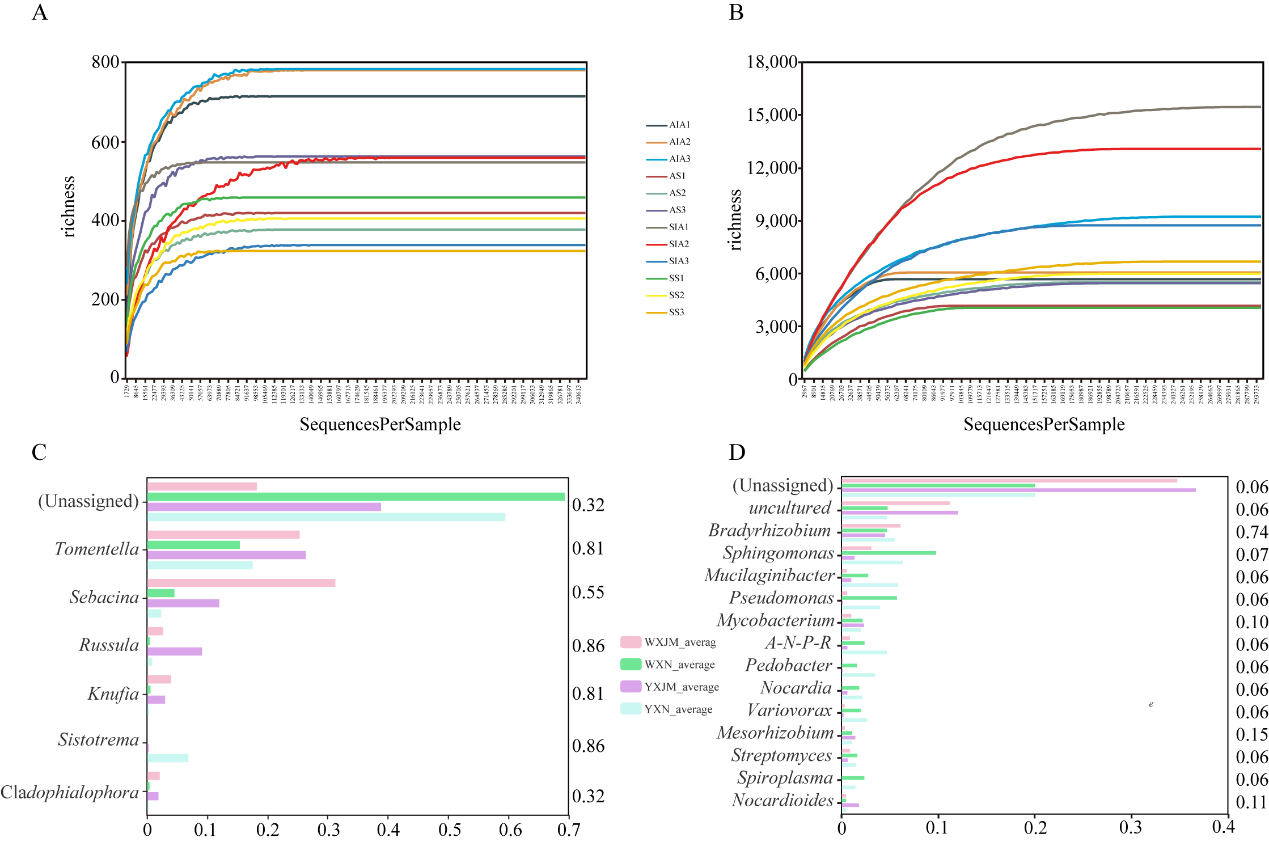
Fig S3.** Dilution curve and species difference between groups. (**A)** Fungal dilution curve; (**B)** Bacterial dilution curve；(**C)** Intergroup species differences in fungi；(D) Species differences between bacterial groups

**Note:** “*A-N-P-R”* in Figure D stands for “*Allorhizobium-Neorhizobium-Pararhizobium-Rhizobium*”

**
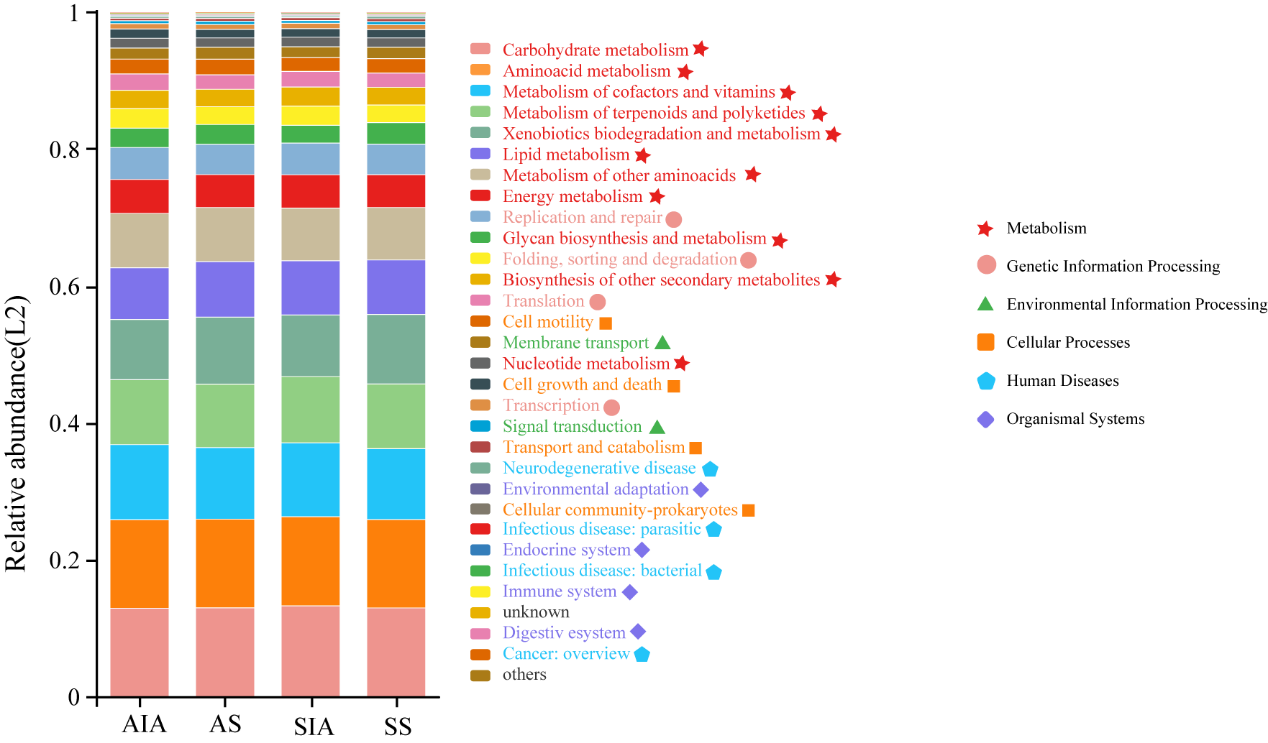
**

**Fig S4.** KEGG pathway abundance composition at the secondary classification level

Note: Different symbols in the Fig. represent different KEGG pathways at the primary classification level.
